# Behavioral control and changes in brain activity of honeybee during flapping

**DOI:** 10.1002/brb3.2426

**Published:** 2021-11-22

**Authors:** Haojia Ding, Jieliang Zhao, Shaoze Yan

**Affiliations:** ^1^ Division of Intelligent and Biomechanical Systems State Key Laboratory of Tribology Department of Mechanical Engineering Tsinghua University Beijing China; ^2^ School of Mechanical Engineering Beijing Institute of Technology Beijing China

**Keywords:** behavior control, electrical stimulation, evoked potential, honeybee

## Abstract

**Introduction:**

Insect cyborg is a kind of novel robot based on insect–machine interface and principles of neurobiology. The key idea is to stimulate live insects by specific stimuli; thus, the flight trajectory of insects could be controlled as anticipated. However, the neuroregulatory mechanism of insect flight has not been elucidated completely at present.

**Methods:**

To explore the neuro‐mechanism of insect flight behaviors, a series of electrical stimulation was applied on the optic lobes of semi‐constrained honeybees. Times of flight initiation, flapping frequency, and duration were recorded by a high‐speed camera. In addition, flapping and steering initiation experiments of the cyborg honeybee were verified. Moreover, series of local field potential signals of optic lobes during flapping were collected, pre‐processed to remove baseline wander and DC components, then analyzed by power spectrum estimation.

**Results:**

A quantitative optimization method and optimal stimulation parameters of flight initiation were presented. Stimulation results showed that the flapping duration differed greatly while the flapping frequency varied with little difference among different individuals. Moreover, there was always a fluctuation peak around 20–30 Hz in power spectral density (PSD) curves during flapping, distinguishing from calm state, which indicated some brain activity changes during flapping.

**Conclusions:**

Our study presented a range of relatively optimal electrical parameters to initiate honeybee flight behavior. Meanwhile, the regularity of flapping duration and flapping frequency under electrical stimulations with different parameters were given. The feasibility of controlling a honeybee's flight behavior by brain electrical stimulation was verified through the flapping and steering initiation experiment of honeybees under semi‐constrained state. PSD fluctuations reflected changes in brain activity during flapping and that those fluctuation characteristics at the specific frequency band could be sensitive determinants to distinguish whether the honeybee was flying or not, which benefits our understanding of honeybee's flapping behavior and furthers the study of honeybee cyborgs.

## Graphical abstract

A series of optimal electrical parameters were presented to control the honeybee's flapping behavior. Meanwhile, flight and turning initiation of honeybees which can move freely was achieved. Furthermore, flapping‐related brain activity changes were explored using power spectra density analysis.

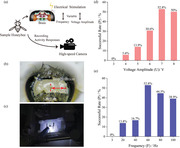

## 1 INTRODUCTION

Insects, which have a unique way of flight way for flapping wings at high speed, can adapt to many kinds of environment, and are thus spread all over the world. Inspired by those unique advantages of insects’ flight ability, researchers have developed bionic robots and micro air vehicles (MAV) (Pines & Bohorquez, 2006; Sane, 2003), even employed the inspiration to the design of aerospace vehicles (Zhang et al., 2019; Liang et al., 2019). Nevertheless, due to the limitations of micromachining methods and the shortcomings of power systems, payloads and navigation technologies, artificial MAVs cannot fly as freely as real insects. (Bermudez & Fearing, 2009; Breugel et al., 2008; Whitney & Wood, 2010). They could only imitate the flying performance of insects to a certain extent, and were far from comparable with insects in terms of flight speed, perception of external conditions, energy supply, environmental adaptability, effective flight distance, and so forth. In recent years, insect cyborg technologies have been developed and have overcome some technical difficulties of MAVs. At the same time, it brought a new challenge, that is, how to determine optimal stimulus parameters and stimulation sites for flight behavior regulation.

Before insect cyborgs came out, researchers had already tried to control live insects’ behaviors by stimulating some specific body parts of insects. Huber performed the electrical stimulation experiment on the insect supraesophageal ganglion by using acute preparations of *Gryllus* and found that the mushroom body and the central body were relevant to higher control of different motions in orthopteran insects (Huber, 1960). Rowell (1963) controlled a series of behaviors such as antennal movements, locomotion, feeding, and sexual behavior of locusts for the first time.

To gain more precise insects’ behavior control results, some more systematic control strategies by stimulation appeared. Electrical stimulation is an effective way to control the flight or walking behaviors of insects based on neuro‐mechanism study and micro‐electronic mechanical systems (MEMS). Stimulating the antenna by electricity is the earliest method to control the insect behavior. Holzer & Shimoyama (1997) groundbreakingly controlled a live cockroach to walk along a black line by stimulating its antenna with an electronic backpack. Similarly, since physical activities of antenna can affect the motion of insects, the electroantennography (EAG) of honeybees under different odors was investigated to analyze the response of samples in order to provide physical support to control live honeybees (Zhao et al., 2020). Furthermore, cyborgs with other functional modules, such as a kind of electrical stimulated cyborg mounted on a sound source localization module, came out (Latif et al., 2016). Vo Doan et al. (2015) demonstrated the achievement of free‐flight thrust control of cyborg beetles (Vo Doan et al., 2015). Based on this research, more flexible motion control with a user‐adjustable walking gait, step length, and walking speed was achieved. (Cao et al., 2016).

An effective method to control the motion of insects, neuron stimulation, was proposed (Bozkurt et al., 2008a). Tsang et al. (2008) developed a flexible electrode array that provided multisite electrical stimulation of an interganglionic bundle of nerve fibers in the moth's abdominal nerve cord. Therefore, the motion of adult moths’ abdomen induced by stimulating the nerve cord would lead to scalable motion in direction during flight (Tsang et al., 2008). Later, a terrestrial cyborg with a neural stimulation system was demonstrated and a system‐on‐chip, which enabled a wireless neuro‐stimulation backpack system with onboard electrodes‐tissue bioelectrical coupling verification, was proposed (Latif & Bozkurt, 2012). Since the physiological mechanism of neural stimulation is not clear, a new brain stimulation protocol for the honeybee‐machine interface was presented. (Zhao et al., 2014). Recently, a functional brain‐to‐brain interface which was more efficient than insect–machine interface was achieved. By using the above method, researchers used orders from the human brain to guide a live cockroach (Li & Zhang, 2016).

Researchers also have been trying to combine neuron and muscle electrical stimulation methods for higher robustness. Sato et al. (2008a) achieved initiation, cessation, and elevation controls of giant beetles (*Mecynorhina torquata*) by stimulating optic lobes. Then, they presented an implantable flight control microsystem including a visual stimulator, a polymide assembly, a microcontroller, and multiple inserted neural and muscular stimulators (Sato et al., 2008b). Further, wireless continuous flight control of beetles with a miniaturized system was achieved (Sato et al., 2009). Their miniature radio system could remotely stimulate free‐flying insects and they found that the coleopteran third axillary muscle was tonically activated ipsilaterally during turns (Sato et al., 2015). In order to improve the robustness of the electrodes‐tissue interface, an Early Metamorphosis Insertion Technology (EMIT), which means placing the control micro‐chip in insects’ pupal stage, was proposed (Bozkurt et al., 2007). Based on EMIT, they developed a radio‐controlled cyborg by neuromuscular activation, used a helium balloon to increase the payload capacity and flight duration, at the same time enabled varies kinds of applications (Bozkurt et al., 2009a). Later, they demonstrated their results towards navigation of flight in moths which instrumented with equipment to gather information for environmental sensing (Bozkurt et al., 2009b). In addition, combining neural and muscular stimulators, Daly et al. (2010) developed a 2.5 mW wireless flight control system for cyborg moths.

Except for electrical stimulation, researchers also have been trying to control insects by micro‐thermal stimulation (Bozkurt et al., 2008b; Visvanathan et al., 2008), chemical stimulation (Chung & Erickson, 2009), and visual stimulation (Van Kleef et al., 2013; Verderber et al., 2014; Zheng et al., 2018). Although tremendous efforts have promoted technical progress more accurately for controlling insects by various stimulation methods, a systemic brain stimulation prototype of insects has not been established yet. Neuro‐mechanism of controlling insects flight by stimulating brain subregion is so vague that accurate flight control technologies cannot be realized at present. In this paper, we put electrical stimulation of different parameters on the optic lobes of honeybees to find out the optimal frequency and voltage amplitude of the specific electrical pulse. At the same time, to explore the optimal parameters and stimulation sites, the research on behaviors' neuro‐mechanism should be furthered. Brain electrophysiological signals analysis is the main research means to explore the neuro‐mechanism of behaviors (Wolpaw et al., 1998). Thus, LFP signals during flapping were recorded and analyzed by power spectral estimation, which will deepen our comprehension towards insect flight control neuro‐mechanism and further the study of accurate flight control of honeybees.

## 2 MATERIALS AND METHODS

### 2.1 Sample preparation

Foraging workers (*Apis melifera carnica*) were captured from honeybee hives (temperature:25°C, humidity: 55%) in the Tsinghua University lab (116.33°E, 40.00°N). Before fixation and anatomy, captured honeybees were foraged with sucrose solution in bottles.

### 2.2 Fixation and anatomy

In the completely constrained state, a scaffold was designed to fix the honeybee's head firmly at an immobilized position to locate the specific brain subregion more accurately. After the sample was tethered to the scaffold, melting beeswax was put on the sample's rostral head, which was handled by an electric tachometer indicator torch. Therefore, the interspace between the scaffold and the rostral head was filled with beeswax. Thus, the honeybee's head was fixed firmly on the scaffold. A rectangle small cuticle was cut from the honeybee's head. Glands and trachea were carefully removed and the brain was then covered with saline.

Under the semi‐constrained state, the pretreatment procedures were consistent with the previous experiments. When the preparation steps were finished, the stimulating electrode was inserted into a pre‐punched hole on the head cuticle, which was about 850 μm below the brain surface. A small amount of beeswax was dripped on the surface of the bee's head cuticle. After the beeswax solidified, a small amount of white glue was coated on the surface of the cuticle. The electrode implantation procedure was completed when the white glue solidified. Here, beeswax was used for isolating the glue from the brain of the honeybee because white glue was water‐soluble and toxic to honeybees.

### 2.3 Electrode implantation and position

The Tungsten electrodes (Kedou Brain‐Computer Technology Co., Ltd, Suzhou, China) were selected as stimulating electrodes due to their electrical conductivity and high strength at μm scale. One end of the stimulating electrodes (insulation side) was fixed on the displacement controller (CTF‐8301B3, Rich life Science Instrument Co., Ltd, Jiangsu, China) and the other end was connected to the isolated pulse stimulator (Model 2100, A‐M Systems, US). After the stimulating electrode was implanted into optic lobes, the reference electrode was placed at the brain surface to form a complete circuit.

**FIGURE 1:**
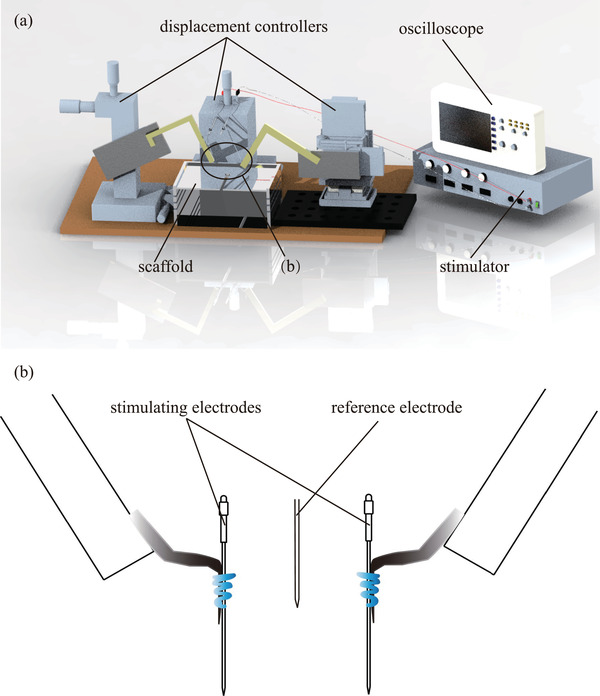
Brain stimulation experiment platform. (a) Schematic diagram of experimental equipment. (b) Diagram of the stimulating electrodes and the reference electrode

**FIGURE 2:**
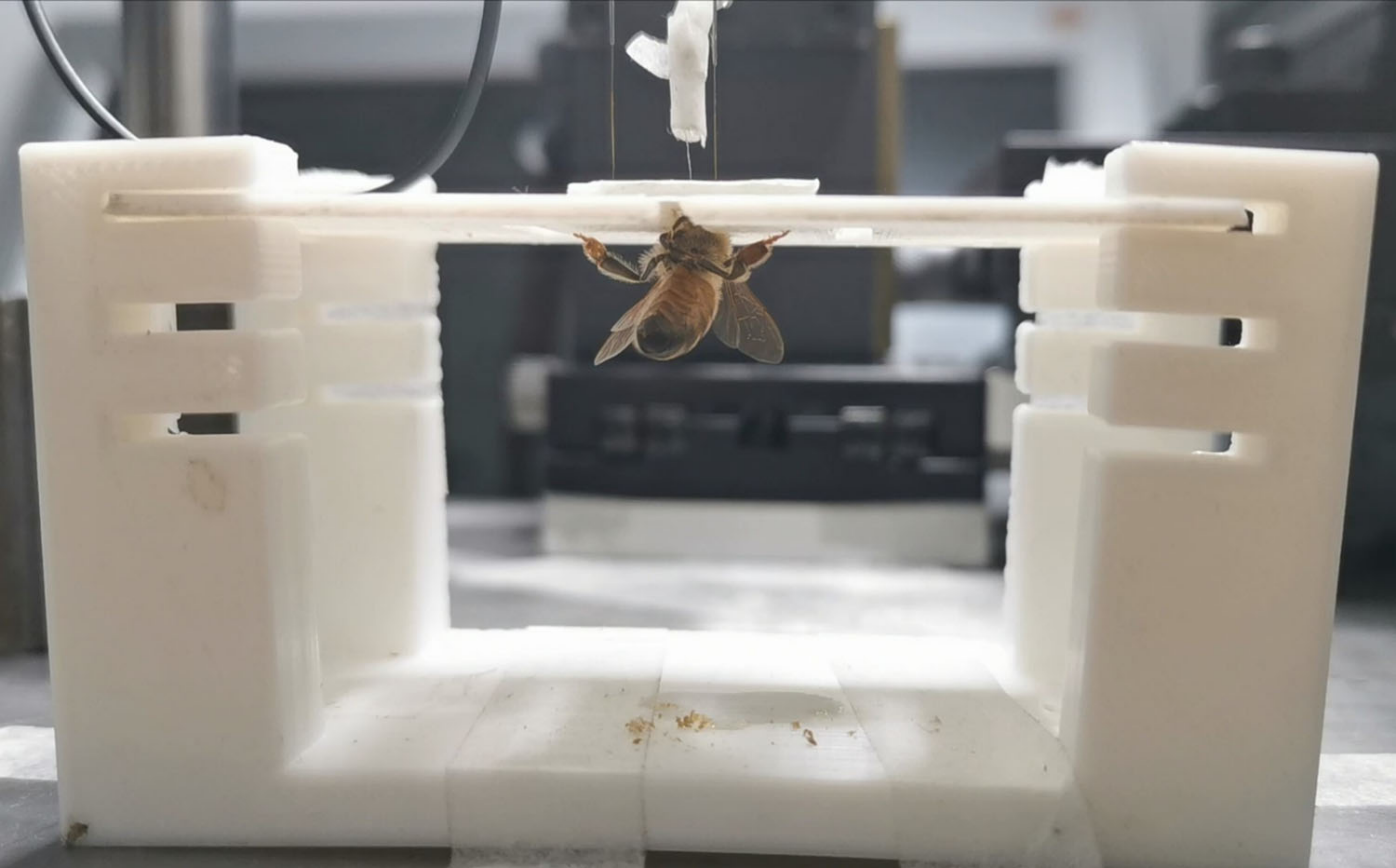
The stimulating electrodes and reference electrode were implanted in optic lobes of the fixed honeybee

**FIGURE 3:**
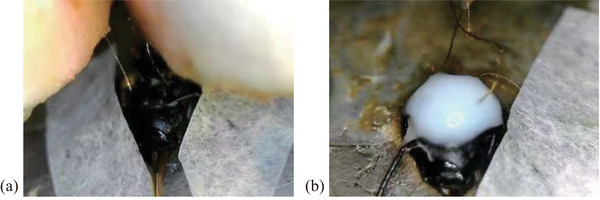
The honeybee with fixed electrodes during the operation. (a) Stimulating and reference electrodes were already fixed. (b) A small amount of white glue was coated on the surface of the cuticle

**FIGURE 4:**
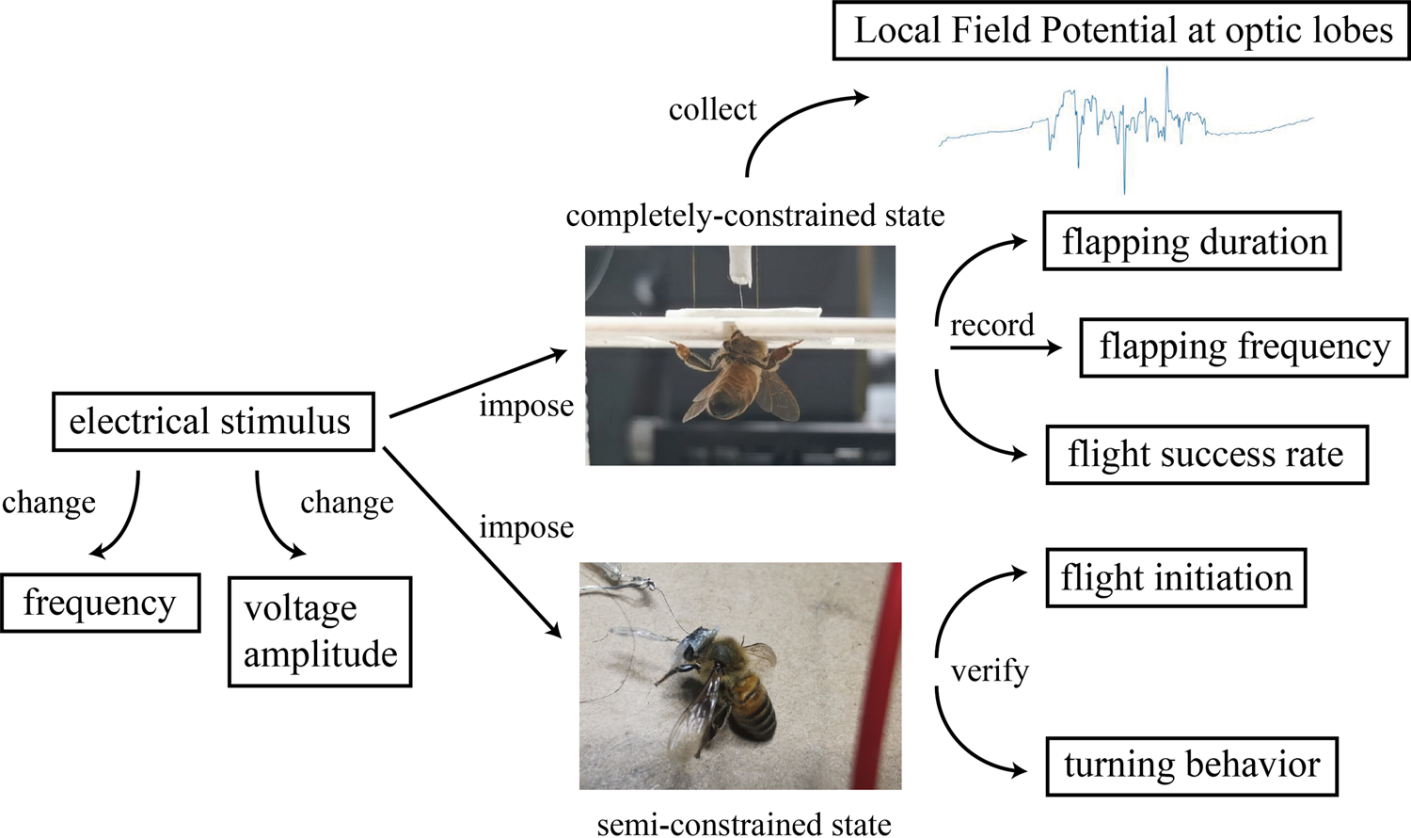
The overall flow path of optimal stimulation parameters exploration experiments and LFP research

The stimulation sites of the optic lobes were positioned by three displacement controllers (as shown in Figure 1). When the stimulating electrode contacted the brain surface, it kept looping it down until it reached the specific depth. Thus, the procedure of electrode implantation was finished. Under the completely constrained state, the sample's state when preparation procedure was completed is shown in Figure 2. Figure 3 shows the electrodes fixation operation under the semi‐constrained state. The overall framework of the experimental design is shown in Figure 4.

### 2.4 Local field potential recording

Under the completely constraint state, the electrical stimulus (with a duty ratio of 50%, voltage amplitude of 7 V, and a stimulus duration of 0.4 s), was imposed on the optic lobes of the samples. At the same time, a series of local field potential (LFP) signals collected from the optic lobe were recorded by the 4‐channel USB acquisition controller (Sytech, Buchenbach, Germany). Since LFP signals were collected from one side of the optic lobes, only channel‐1 of the 4‐channel USB acquisition controller was used. Data was collected by the software “EagPro” in real time.

## 3 RESULTS AND DISCUSSION

### 3.1 Optimal electrical stimulation parameters exploration

The stimulus of rectangular biphasic pulse (duty ratio: 50%) was generated by an isolated pulse stimulator and was used for stimulation, the duration of which was 0.4 s. A series of frequency and voltage amplitude parameters were designed. The principle of selecting the upper limit of the parameter range is to ensure the honeybees’ high activity. The combinations of experimental parameters are shown in Table 1. These stimuli combinations were applied to the optic lobes of the samples to record their corresponding behavior response in order to narrow the range of optimal stimulation parameters.

**TABLE 1:** Combinations of frequency and voltage amplitude used for stimulation

	Voltage amplitude ***U***_***i***_ (V)							
Frequency ***F***_***i***_ (Hz)	***U***_1_(0.3)	***U***_2_(0.5)	***U***_3_(0.9)	***U***_4_(1.6)	***U***_5_(2.7)	***U***_6_(4.7)	***U***_7_(8.1)	***U***_8_(24.0)
***F***_1_(1)	***F***_1_ ***U***_1_	***F***_1_ ***U***_2_	***F***_1_ ***U***_3_	***F***_1_ ***U***_4_	***F***_1_ ***U***_5_	***F***_1_ ***U***_6_	***F***_1_ ***U***_7_	***F***_1_ ***U***_8_
***F***_2_(3)	***F***_2_ ***U***_1_	***F***_2_ ***U***_2_	***F***_2_ ***U***_3_	***F***_2_ ***U***_4_	***F***_2_ ***U***_5_	***F***_2_ ***U***_6_	***F***_2_ ***U***_7_	***F***_2_ ***U***_8_
***F***_3_(10)	***F***_3_ ***U***_1_	***F***_3_ ***U***_2_	***F***_3_ ***U***_3_	***F***_3_ ***U***_4_	***F***_3_ ***U***_5_	***F***_3_ ***U***_6_	***F***_3_ ***U***_7_	***F***_3_ ***U***_8_
***F***_4_(30)	***F***_4_ ***U***_1_	***F***_4_ ***U***_2_	***F***_4_ ***U***_3_	***F***_4_ ***U***_4_	***F***_4_ ***U***_5_	***F***_4_ ***U***_6_	***F***_4_ ***U***_7_	***F***_4_ ***U***_8_
***F***_5_(100)	***F***_5_ ***U***_1_	***F***_5_ ***U***_2_	***F***_5_ ***U***_3_	***F***_5_ ***U***_4_	***F***_5_ ***U***_5_	***F***_5_ ***U***_6_	***F***_5_ ***U***_7_	***F***_5_ ***U***_8_
***F***_6_(320)	***F***_6_ ***U***_1_	***F***_6_ ***U***_2_	***F***_6_ ***U***_3_	***F***_6_ ***U***_4_	***F***_6_ ***U***_5_	***F***_6_ ***U***_6_	***F***_6_ ***U***_7_	***F***_6_ ***U***_8_
***F***_7_(600)	***F***_7_ ***U***_1_	***F***_7_ ***U***_2_	***F***_7_ ***U***_3_	***F***_7_ ***U***_4_	***F***_7_ ***U***_5_	***F***_7_ ***U***_6_	***F***_7_ ***U***_7_	***F***_7_ ***U***_8_
***F***_8_(1000)	***F***_8_ ***U***_1_	***F***_8_ ***U***_2_	***F***_8_ ***U***_3_	***F***_8_ ***U***_4_	***F***_8_ ***U***_5_	***F***_8_ ***U***_6_	***F***_8_ ***U***_7_	***F***_8_ ***U***_8_

**FIGURE 5:**
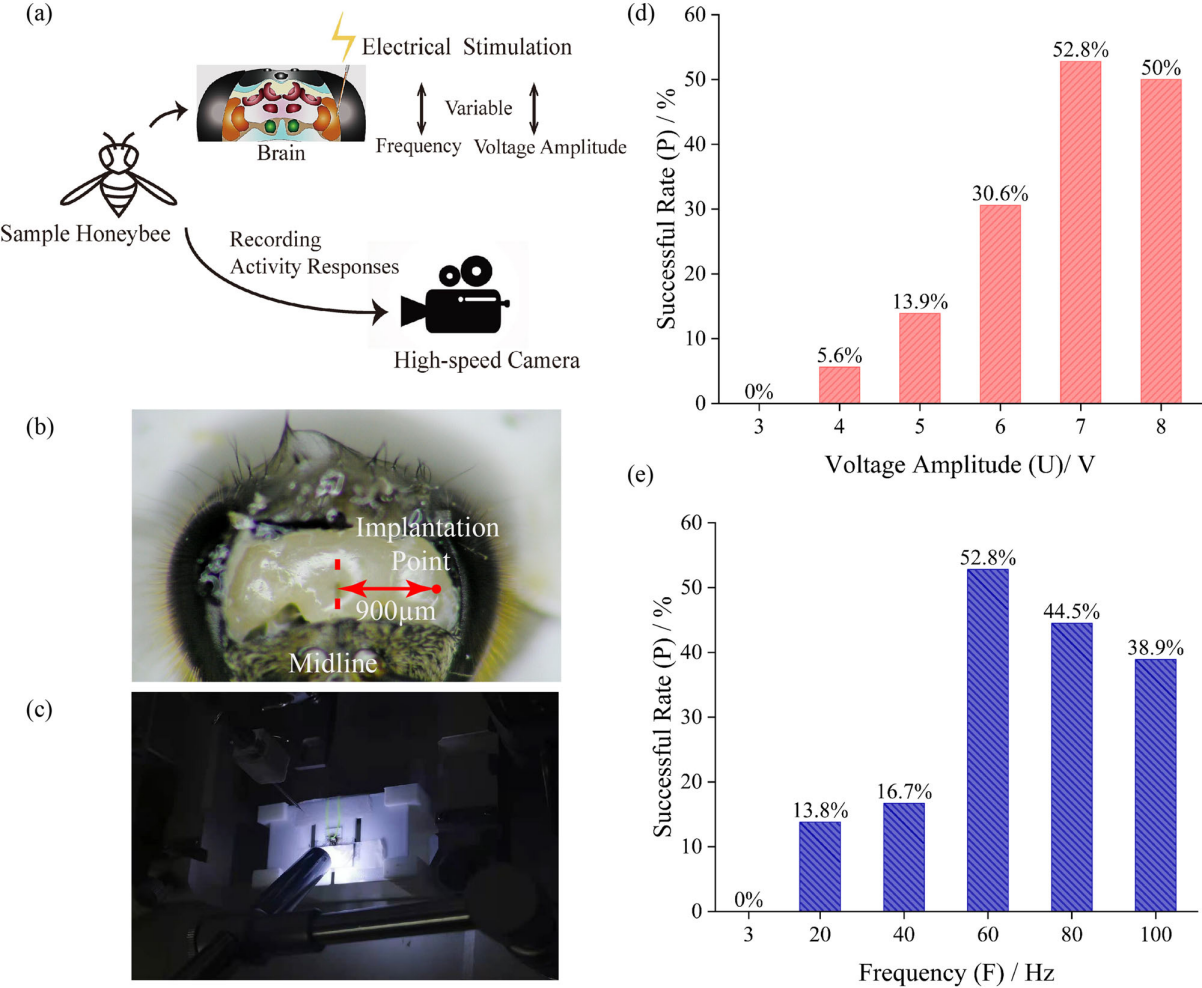
Overall stimulation experimental procedures and statistic results of optimal electrical stimulation parameters exploration. (a) Sample honeybees’ optic lobes were stimulated by electrical stimuli of variable parameters of frequency and voltage amplitude. At the same time, activity responses were recorded by the high‐speed camera. (b) The sample honeybee's brain surface and the electrode implantation point. (c) The tethered honeybee waiting for electrode implantation. (d) The flight initiation successful rate (*P*) under different stimulation parameters of frequency and voltage amplitude

From the data, we found that when the frequency was between ***F***_3_****** and ***F***_5_, voltage amplitude was between ***U***_5_ to ***U***_7_, *** *** the success rate of flapping initiation response could reach 80%. Based on these results, the range of frequencies were narrowed to 3, 20, 40, 60, 80, and 100 Hz. Meanwhile, the range of voltage amplitude were narrowed to 3, 4, 5, 6, 7, and 8 V. Then, these stimuli combinations were applied to optical lobes of eight honeybees to observe their reactions. After the electrodes were implanted into the brain, the isolated pulse stimulator was set to expected stimulating parameters. Then, push the start button of “single stimulus”; thus, the stimulus will be applied on the sample's optic lobes. The activities of samples were observed at the same time. When the sample starts to flap, turn on the high‐speed camera (Phantom, M110, Vision Research, Wayne, NJ) to record its activity and flapping duration. The overall procedures are shown in Figure 5a. Figure 5b shows a honeybee's brain surface after the head cuticle was removed and the electrode implantation point and Figure 5c shows the electrodes implantation procedure.

When stimulations were in low density, which meant the amplitude and frequency were both low, only legs stretched and contracted, or wings slightly evoked upstroke and downstroke, but flight behavior did not happen. To analyze the effect of voltage amplitude, we recorded the times of flight initiation among eight tethered honeybees when electrical stimulations with different amplitudes were applied. Experimental data were summarized in Figure 5d,e. The successful rate (*P*) is defined as the ratio of the times of flight initiation and the overall experimental times. Results showed that when the voltage amplitude (*U*) was lower than 7 V, *P* among 12 tethered honeybees increased as *U* increased. While *U* was higher than 7 V, *P* seemed to be a downward trend. These results showed that there was an optimal parameter of *U* around 7 V. Also, to analyze the effect of frequency, the times of flight initiation were recorded when rectangular biphasic pulses with different frequencies were applied to honeybees. When the frequency (*F*) was lower than 60 Hz, *P* increased with the increase of the frequency. Conversely, *P* decreased when *F* was higher than 60 Hz, as shown in Figure 5e. This result indicated that there was an optimal frequency parameter of around 60 Hz when such a rectangular biphasic pulse, mentioned above, was applied on honeybees’ optic lobes. In general, a stimulus used for initiating honeybees flight behavior with parameters, mentioned above, the success rate Pcan be guaranteed when voltage amplitude (*U*) is around 7 V and frequency(*F*) is around 60 Hz.

**FIGURE 6:**
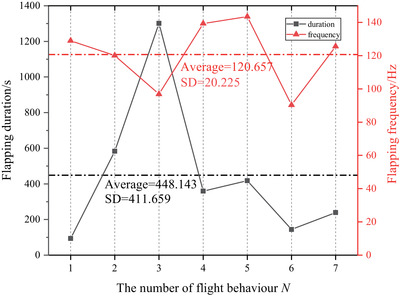
The correlation analyzation results between flapping frequency and flapping duration. The average value of the flapping duration was 448.143 s and Standard Deviation (SD) was 411.659, which indicated that the flapping duration tended to be random. While the average value of flapping frequency was 120.657 s, SD = 20.225, which indicated that a certain electrical stimulus perhaps had some inherent connections with flapping frequency of honeybee

Furthermore, in order to explore the effect of artificial electrical stimulation on flight behavior, the pulse with the frequency of 200 Hz, voltage amplitude of 5 V, duty ratio of 40%, stimulus time of 1 s was applied to nine honeybees. When these samples started to flap, the flapping frequency and the flapping duration were recorded by a high‐speed camera. The experimental data is shown in Figure 6. The mean values of flapping frequency and the flapping duration were 120.6 Hz and 448 ms, respectively. When the honeybee was stimulated, the flapping frequency varied with little difference but huge differences in flapping duration. This indicated that when honeybees’ flight behavior was controlled artificially, the flapping frequency was always roughly consistent, and the flapping duration varied due to the individual difference of honeybees. These results coincided with a previous study (Bao et al., 2011), that is, the flapping duration varied widely due to the activity of honeybees.

In order to explore the correlation between the flight duration and flight frequency of honeybees, the correlation coefficient and covariance were calculated. The calculation results have shown that, the correlation coefficient between them was −0.35, and the covariance was −2505.15. The results showed that there was a negative correlation between flight duration and flight frequency, but the correlation was weak. However, the value of the covariance was large, indicating that the dispersion between the two sets of data was large. And it also proved that there was no obvious correlation between them. In summary, the flight duration and flight frequency can be regarded as two independent parameters during artificially controlled flight, which basically do not interfere with each other. The statistical results are shown in Figure 6.

### 3.2 Flight validation of honeybee cyborgs by electrical stimulation

Under the completely constrained state, the stimulation parameters for flight initiation were at a frequency of 100 Hz, a duty cycle of 40%, and a stimulation duration of 1 s, with bipolar pulse. The stimulation parameters for steering initiation were at a frequency of 100 Hz, a duty cycle of 40%, and a stimulation duration of 1 s, with unipolar pulse. During the experiment, the high‐speed camera was used to record behaviors of the honeybee.

In the experiment, beeswax was used to fill the exoskeleton and white glue to fix electrodes, which could retain the honeybee's activity for a long time. Beyond that, the electrodes implantation procedure did not affect the bee's motor function. The honeybee could crawl and fly normally even if the implantation was finished. Not only that, the survival time of the sample honeybees could reach 24 h.

**FIGURE 7:**
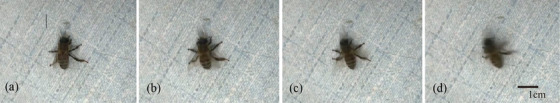
The sample honeybee after stimulation started to flap. From left to right: (a) *t* = 0.5 s, (b) *t* = 1 s, (c) *t* = 1.5 s, (d) *t* = 2 s. Over time, the sample's flapping frequency became faster

**FIGURE 8:**
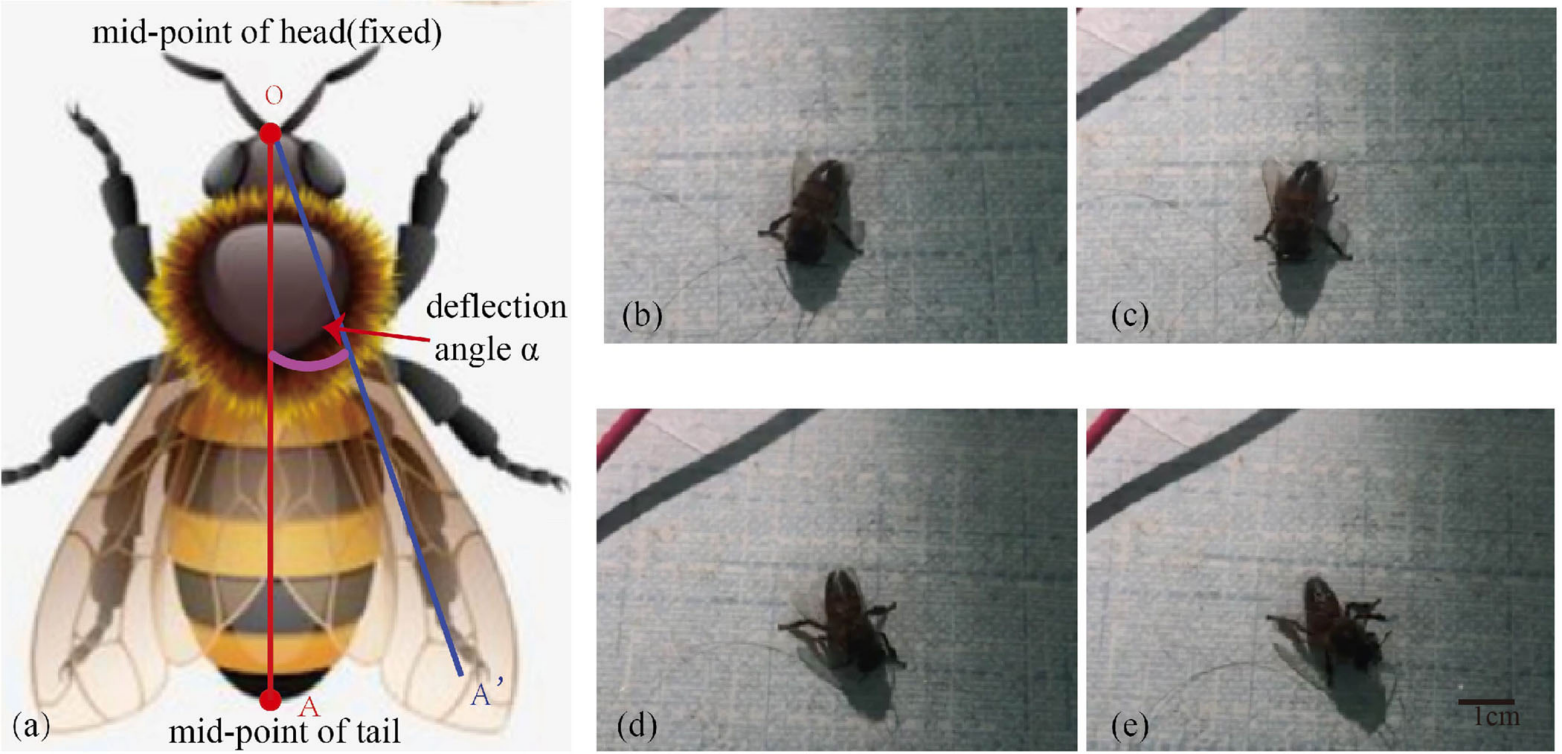
Steering initiation experiments of honeybees. (a) Schematic diagram of deflection angle *α*. (b–e): *t* = 0 s, *t* = 1 s, *t* = 2 s, *t* = 3 s. Point O is fixed and the line between the midpoint O of the bee's head and the midpoint A of the tail serves as a deflection line. The left side angle between OA and L is defined as the deflection angle, which can be measured as 0°, 5°, 47°, and 63°, respectively

Experimental results proved that electrical signals could trigger the honeybees to fly. Figure 7 shows that the sample honeybee after electrical stimulation started to flap and intended to fly. There was a good correlation between times of electrical stimulation and times of flight initiation. At the same time, the electrical signal could induce the direction adjustment of the honeybee's trunk. When the unipolar stimulus was applied, the honeybee showed an obvious unilateral tendency or turning behavior (as shown in Figure 8). Its behavior had good symmetry along with the polarity of stimulation on optic lobes from the left side to the right.

### 3.3 Local field potential analysis

The original LFP signals were imported to the software “MATLAB”, then preprocessed and analyzed by power spectral density (PSD) estimation of the Burg method. In addition, procedures of electrodes implantation were the same as mentioned above. The only difference was that stimulating electrodes were replaced by the recording electrode (Kedou Brain‐Computer Technology Co., Ltd, SuZhou, China). The general flow of the LFP processing is described as follows. After DC components were removed, empirical mode decomposition (EMD) was used for baseline wander correction. Then PSD analysis based on AR model was used for feature extraction.

#### 3.3.1 Signal preprocessing

The LFP signals were relatively weak, and the frequency was low. The baseline wander frequency was mainly concentrated around 0.7 Hz. Thus, it is necessary to remove the baseline wander to prevent the interference in low‐frequency components. At the same time, the DC components of original signals interfere with the identification of the dominant frequency and other characteristics in PSD analysis. Therefore, the DC components were removed by fast Fourier transform (FFT) and inverse fast Fourier transform (IFFT) method.

**FIGURE 9:**
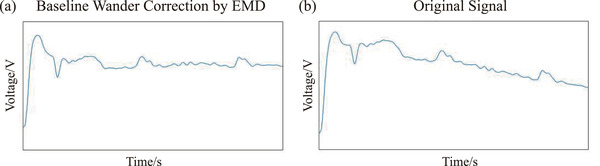
The signal waveform comparison before and after EMD baseline wander correction. (a) The waveform after EMD baseline wander correction. (b) The original signal waveform

Here, EMD was selected to correct the baseline wander. The method decomposes the original signal into a series of intrinsic mode functions in the order of high frequency to low frequency, and then combines the IMF components according to the frequency characteristics of each intrinsic mode function (Qi et al., 2010). Since baseline wander components are concentrated in low frequency, the low‐frequency IMF components should be removed from the original signal, then reconstructed in order to remove the baseline wander. As shown in Figure 9, the signal waveform was improved obviously after baseline wander correction.

#### 3.3.2 PSD analysis results

The LFP signal whose amplitude changes over time is converted into a spectrum of LFP power changes with frequency; thus, the distribution and rhythm changes in different frequency bands. Compared with classical methods, modern PSD estimation methods effectively overcome shortcomings such as the low resolution and poor variance performance. The modern method, called Burg method based on AR model, was used for estimating the power spectrum in this paper. LFP signals analyzed by Burg method are divided by the whole frequency domain into a sum of frequency components by wavelet decomposition in order to provide information on the LFP signal power at a narrow frequency range (Akin & Kiymik, 2000). In case that the analyzed signal is short, Burg method can ensure a stable AR model and achieve efficient calculation. In addition, PSD results obtained under this premise are very close to true values.

**FIGURE 10:**
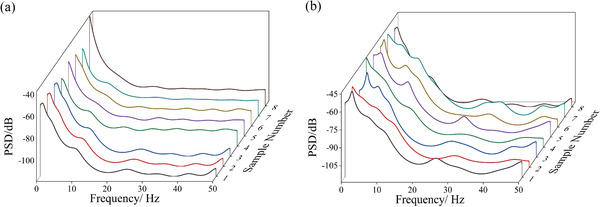
PSE results of eight flapping state and calm state LFP signals based on the Burg method

**FIGURE 11:**
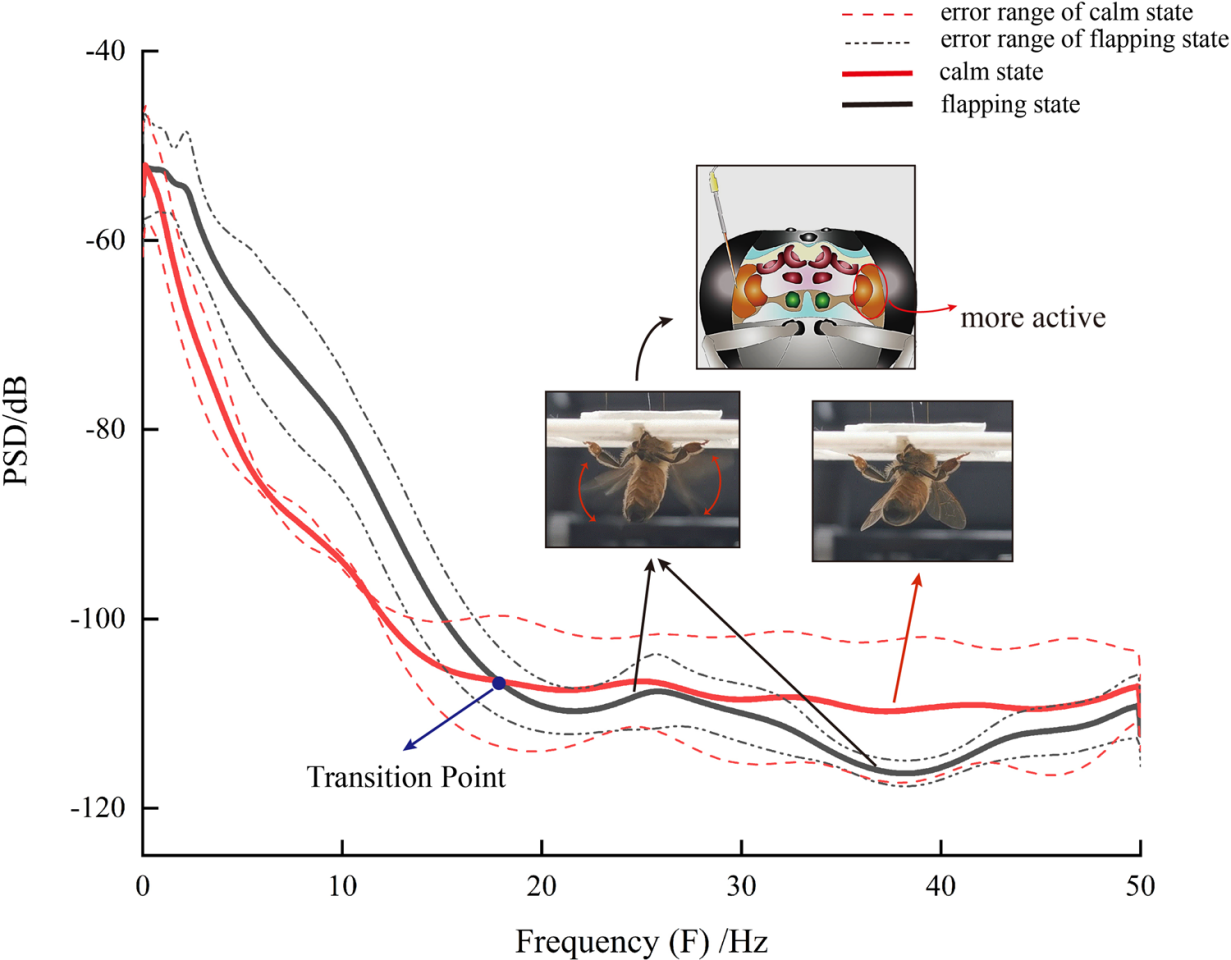
Average PSD results in flapping states and calm states. The transition point was added to explain brain activity changes related to LTM

In this paper, the number of DFT points used in Burg method was 1024 and the order of the autoregressive model was 15. Due to the limitation of space, Figure 10 shows the power spectrum estimation (PSE) results of eight signals in flapping state and calm state, respectively. It can be observed that during flight, the change trend of power over time presented a high degree of similarity. In terms of power spectrum value, as the frequency increased, the power spectrum value in low frequency band generally showed a decreasing trend, and then became flat in higher frequency band. The upper and lower bounds of the value were basically stable. Moreover, there was always a power spectrum value peak at about 20–30 Hz. The amplitude of fluctuations was approximately the same although the peaks were not extremely high. Figure 9 shows the comparative PSD results between the honeybee's LFP signal in flapping state and calm state. Comparing the two power spectrum curves, it can be found that the curve during flight fluctuated more than that of bees in calm state. In the low frequency band, the curve in calm state was flatter; obviously, while there were several small fluctuations in flapping state. This indicated that the change of LFP power in frequency domain during flight was more complex. In terms of power spectrum value, the difference between the upper and lower bound value was not obvious but the energy during flight in lower frequency (around 0–15 Hz) was higher than in calm state. This phenomenon perhaps has some connections with the labor division and cooperation of different brain subregions during flapping. Subsequently, the average PSD value and the error of the eight samples under flapping and calm state respectively mentioned above were calculated, as shown in Figure 11. It can be seen that there was a spectral peak between 20 and 30 Hz in the average PSD curve, which was roughly the same as the conclusion obtained from Figure 10. In addition, observing the results of the error bar, the similarity of PSD curves at higher frequency (greater than 20 Hz) was higher, while the differences at lower frequency were relatively large, which meant greater fluctuations and complication of energy activities in higher frequency bands. In summary, by analyzing the PSD analysis results of 40 samples, features of second‐order spectrum and conjectures of changes in brain activity during flapping were proposed. Whether the honeybee was in flapping state or calm state can be distinguished by the characteristics of PSD mentioned above.

From the aspect of rhythm power changes, the power increase of lower frequency band (lower than transition frequency) and the power decrease of higher band (higher than transition frequency) can be observed if power in a flapping state compared with a calm state. According to the researches performed on humans, selective suppression of alpha power in different sub‐bands and some effects of visual stimulation are closely related to the attentional and semantic memory demands (Klimesch et al., 1997a, 1997b). As for humans, semantic memory demands always means that the meaning of the perceived information is extracted to be stored in semantic long‐term‐memory system (LTMS). The similar power change results can be conjected to suggest that encoding of sensory information during flight for extracting the meaning of perceived information which is stored in LTMS is a powerful factor of the trim control ability of honeybees.

## 4 CONCLUSIONS

The primary technical obstacle of insect cyborgs is that the control accuracy is hard to improve. Here, we demonstrated an electrical stimulation method to control the flight behavior of tethered honeybees. The regularity of the success rate of flight initiation to the electrical stimulation parameters of voltage amplitude and frequency was also researched. Meanwhile, the regularity of flapping frequency and flapping duration among different individuals was explored. It was found that flapping frequency was almost consistent among different individuals while flapping duration differed greatly, which was instructive to control fine parameters of flight behaviors. By implanting two electrodes into optic lobes with certain electrical stimulation, the flight initiation of tethered honeybees could be controlled. In order to achieve higher flight initiation success rate, the exploration of optimal stimulation parameters of voltage amplitude and frequency should be focused. Furthermore, the threshold value of flight initiation should be explored.

In experiments of semi‐constrained state, the initiation of flapping and steering of honeybees has been successfully achieved. By measuring the deflection angle defined, the turning behavior control has been achieved. In order to improve the success rate of flapping and steering initiation, a deep exploration to find better electrical stimulation parameters should be carried out. Thus, the flight and turning behaviors of honeybees can be regulated more precisely. In addition, the fixation method of the electrodes in the semi‐constrained state can also be improved to achieve better stability and more convenient operability.

Furthermore, several LFP analysis methods were applied to explore the energy characteristics of the LFP signals during flight. Analysis results indicated that the distribution rules and values of PSD in frequency domain had a high degree of similarity and there was always a fluctuation peak at around 20–30 Hz. This characteristic could be used as the distinguishing condition for judging whether the tested honeybee was in flapping state. The PSD curves during flight fluctuated more greatly than in calm state, which indicated the brain activity during flight had more complex state change modes and dynamic behaviors. In addition, from the aspect of rhythm power changes, it is suggested that power changes reflected the encoding of sensory information combined with extraction of perceived information from LTMS. All these brain activities contribute to the trim flight control ability of honeybees.

Although insect robots have many advantages over traditional bionic robots and have achieved certain research results, there are still many problems in neural regulation mechanism, such as the coupling of insect–machine interface and the design of micro‐control system. Here, mechanism of neural regulation by electrical stimulation was explored tentatively and some parameters to achieve more accurate behavior control were presented. Brain activity during flight of honeybees was analyzed which further our comprehension of honeybee's flight control neuro‐mechanism.

## CONFLICT OF INTEREST

The authors declare no conflict of interest.

## AUTHOR CONTRIBUTIONS

Haojia Ding performed the experiments, analyzed, and wrote the manuscript. Haojia Ding and Jieliang Zhao conceptualized the work. Shaoze Yan and Jieliang Zhao critically revised the manuscript.

### PEER REVIEW

The peer review history for this article is available at https://publons.com/publon/10.1002/brb3.2426

## Data Availability

The data generated and analyzed in this study are available from the corresponding author upon reasonable request.
